# Deep-Pneumonia Framework Using Deep Learning Models Based on Chest X-Ray Images

**DOI:** 10.3390/diagnostics10090649

**Published:** 2020-08-28

**Authors:** Nada M. Elshennawy, Dina M. Ibrahim

**Affiliations:** 1 Computers and Control Engineering Department, Faculty of Engineering, Tanta University, Tanta 31733, Egypt; Nada_elshennawy@f-eng.tanta.edu.eg; 2 Department of Information Technology, College of Computer, Qassim University, Buraydah, Saudi Arabia

**Keywords:** detecting pneumonia, deep learning, CNN, LSTM, chest X-ray image

## Abstract

Pneumonia is a contagious disease that causes ulcers of the lungs, and is one of the main reasons for death among children and the elderly in the world. Several deep learning models for detecting pneumonia from chest X-ray images have been proposed. One of the extreme challenges has been to find an appropriate and efficient model that meets all performance metrics. Proposing efficient and powerful deep learning models for detecting and classifying pneumonia is the main purpose of this work. In this paper, four different models are developed by changing the used deep learning method; two pre-trained models, ResNet152V2 and MobileNetV2, a Convolutional Neural Network (CNN), and a Long Short-Term Memory (LSTM). The proposed models are implemented and evaluated using Python and compared with recent similar research. The results demonstrate that our proposed deep learning framework improves accuracy, precision, F1-score, recall, and Area Under the Curve (AUC) by 99.22%, 99.43%, 99.44%, 99.44%, and 99.77%, respectively. As clearly illustrated from the results, the ResNet152V2 model outperforms other recently proposed works. Moreover, the other proposed models—MobileNetV2, CNN, and LSTM-CNN—achieved results with more than 91% in accuracy, recall, F1-score, precision, and AUC, and exceed the recently introduced models in the literature.

## 1. Introduction

Pneumonia affects the lungs and causes about 18% of all deaths in children under five years old. Additionally, about two billion people worldwide suffer from pneumonia every year, and death can occur if action is not taken. Early diagnosis of pneumonia is a vital matter [1,2]. Therefore, rapid diagnosis by an expert radiologist using chest X-rays is required to avoid misdiagnosis. Chest X-rays are the most common and cheapest way to detect pneumonia [3,4]. Likewise, there is a shortage of radiologist experts, especially in low-resource countries and in rural regions, causing long waits for diagnoses, which increases the death rate. Because of the nature of chest X-ray image analysis, pneumonia diagnoses by X-ray images are often unclear and can be confused with other diseases that have similar features, such as opacity, cavity, and pleural effusions. Thus, chest X-rays cannot be as easily used for detecting diseases [5]. Accordingly, many computer-aided diagnosis (CAD) systems and computer algorithm diagnostic tools have been proposed by researchers for X-ray image analysis; these proposed systems help radiologists discover various types of chest X-ray pneumonia immediately after acquisition. Recently, various biomedical problems, such as skin cancer detection, brain tumor detection, and breast cancer detection are using solutions based on Artificial Intelligence (AI) approaches [6,7,8,9] as handcrafted techniques, deep learning, and machine learning techniques. As a matter of fact, deep learning is a subsection of AI and machine learning that utilizes multi-layered artificial neural networks to provide the latest technology in many topics, such as speech recognition, language translation, and others. There is a difference between traditional machine learning techniques and deep learning in that the latter can automatically learn representations from data, such as videos, images, or text, without entering manually coded rules or direct human intervention. Their architectures are highly flexible, and thus can learn immediately from the data and raise their predictive accuracy by providing it with more data [10]. The main objective of this work was to develop a deep learning framework to automatically diagnose pneumonia using chest X-ray images and to classify the result as normal cases or pneumonia cases, which will help in quickly and easily diagnosing the disease.

The rest of this paper is organized as follows. Section 2 illustrates reviews on recent related works. In Section 3, the background of deep learning algorithms is presented. Our proposed Deep-Pneumonia framework is demonstrated in Section 4. Section 5 presents the proposed four CNN architectures. The methodology and the experimental results obtained from our proposed models are discussed in Section 6 and Section 7, respectively. Finally, conclusions and future work are presented in Section 8.

## 2. Related Works

Several methods have been introduced in the literature to help in detecting pneumonia using chest X-ray images. Some of these methods use handcrafted feature extraction techniques along with a machine learning algorithm as a classification technique, whereas others use deep learning techniques for feature extraction and classification [11]. These methods have changed the parameters of deep layered CNNs for pneumonia detection that can be used to obtain high accuracy in disease detection. The authors in [12] used logistic regression as a baseline model for pneumonia detection using X-rays. The Area Under the Curve (AUC) of logistic regression does not produce a good result. They used a 121-layer dense convolutional network (DenseNet) to achieve a better result. An Adam optimizer was used to train the network. The AUC in the model was 0.609 in pneumonia detection, which is a little better than logistic regression (AUC 0.60), and they clarified that the X-ray images with pneumonia are present in only 1% of the dataset.

The researchers in [13] built a model that diagnoses pneumonia with high accuracy. The ChexNet contains a 121-layer CNN that analyses the chest X-ray image and determines the likelihood of pneumonia by classifying the image bilaterally (presence or absence) and locating it via a thermal map. They used a dataset (ChestX-ray14) provided by Wang et al. [14]. Due to the difficulty of diagnosing pneumonia, the results of the model (ChexNet) were compared with four radiologists based on F1. A F1 score of 0.435 was obtained for the ChexNet model, and this exceeds that of the average radiologist (0.387). The researchers in [13] faced difficulties—the front pictures were not clear, and access to the patient’s file was not allowed.

The RSNA dataset provided by Kaggle was used in [15], and consists of 26,684 chest X-rays, 6000 of which were from patients with pneumonia, and 20,000 of which were from patients without pneumonia. The image file type was changed to PNG and resized to reduce runtime. A sequential CNN model with RGB images was introduced. They used maximum pooling to achieve the highest pixel from an area of interest, and then flattened the result. Their data were split into Model 1, Model 2a, and Model 2b, and they then used the same introduced models with various training data. These models varied in their required outputs: Model 1 was used to classify the data as pneumonia or without pneumonia, Model 2a was used to classify the data as normal or opaque, and Model 2b used the image classified by Model 2a as an input and re-classified as opacity or pneumonia. Model 1 achieved an accuracy of 78.5%, Model 2a, 68.5%, and Model 2b, 69.9%.

Similarly, the authors in [16] used the RSNA dataset provided by Kaggle to implement two architectures: mask-RCNN and a residual network. It was used to create computer-aided detection to detect pneumonia. In the residual network, they used residual mapping to solve the overfitting. After each convolution and activation, they used batch normalization. They then merged binary cross-entropy for the loss function and the Intersection over Union (IoU). To decrease the amount of parameters, a pooling block was used. The Mask Regional CNN was used to localize objects using a bounding box. It was composed of two stages: Region Proposal Network (RPN) and RoI-align. They used the bottom-up and up-extraction path using the Feature Pyramid Network (FPN) to extract the features. The residual network had a confidence level greater than 0.7 and an accuracy of 85.60%, and Mask-RCNN had a confidence level of 98% and an accuracy of 78.06%.

In Li et al.’s work [17], an algorithm consisting of three parts for pneumonia detection was proposed: CXR image preprocessing, lung Region of Interest (ROI) segmentation with transfer learning, and an automatic detection model for pneumonia based on a CNN. In medical image segmentation tasks, the U-net model was shown. This model was used to segment ROIs from CXR images, achieving 97.7% and 97.1% segmentation accuracy with respect to the Montgomery and JavaScript Runtime (JSRT) datasets, respectively. SENet design was used to improve the full CNN architecture. The introduced detection models used were RetinaNet and Mask R-CNN. The CXR image dataset was obtained from the RSNA pneumonia detection challenge. There were 8964 pneumonia-labeled CXR images, and the remaining 20,025 were non-pneumonia CXR images. Three thousand CXR images were used as test sets in the competition. The accuracy of Mask R-CNN was 0.183, and that of RetinaNet was 0.225.

RetinaNet and Mask R-CNN models were also used in [18]. Since the FPN produces multi-scale feature maps with greater quality information than the default, the FPN base was used as the backbone of both models. They used a publicly available RSNA Pneumonia Detection Challenge dataset that consists of 26,684 unique chest X-rays. There were three classes of labels: normal, which was 29% of all images, no lung opacity/not normal, 40%, and lung opacity, 31%. The images were split into training (25,684) and testing (1000) images in the first stage of the competition. During the second stage, the training was 2684, and the testing was 3000. In their experiments, the training images were divided into two parts: actual training, 90%, and validation, 10%. The final model and the RetinaNet and Mask R-CNN models were implemented in a Keras framework. The results for RetinaNet, Mask R-CNN, and the combined model measured in terms of mean average precision at Stage 1 was, for RetinaNet, 0.192, for Mask R-CNN, 0.169, and for the combined model, 0.199. At Stage 2, RetinaNet was 0.202, Mask R-CNN was 0.165, and the combined model was 0.204.

In [19], the authors presented two CNN architectures—one with a dropout layer and another without a dropout layer. Both CNNs consisted of a convolution layer, a maximum pooling layer, and a classification layer. A series of convolution and maximum pooling layers acted as a feature extractor that was divided into two parts. The first part consists of two convolution layers with 32–32 units, each along with a max-pooling layer of size 3×3 and a Rectified Linear Unit (ReLU) activator. The second part had two convolution layers with 64 and 128 units, respectively, along with a maximum pooling layer of size 2×2 and an ReLU activator. ReLU is a popular activation function that was generally used in neural networks, especially in CNNs. The ReLU layer introduced nonlinearity into the model. The results of the testing accuracy for the four modules were 90.68%, 89.3%, 79.8%, and 74.9%.

For classifying normal and pneumonia patients using chest X-ray images, four common, CNN-based, deep learning techniques were trained and tested in [1]. These algorithms were DenseNet201, ResNet18, SqueezeNet, and AlexNet. The results showed that DenseNet201 outperforms the other three. The accuracy, precision, and recall values of classifying pneumonia and normal images, viral and bacterial pneumonia images, and only normal images were (98%, 97%, and 99%), (95%, 95%, and 96%), and (93.3%, 93.7%, and 93.2%), respectively. The literature models and their results are summarized in Table 1.

Other researchers have used performance metrics, as in [12], where only Area Under the Curve (AUC) was used, in [13], where only F1-score was used, and in [19], where only the accuracy metric was used. Moreover, in [15,16,17,18], accuracy and other metrics were used. However, the author in [1] is the only one that has used all performance metrics, as in our model. Additionally, our models exceed the others in accuracy and all other performance metrics.

## 3. Background of Deep Learning Algorithms

### 3.1. Convolutional Neural Networks (CNNs)

In recent years, the use of deep learning in clinical diagnosis and medical images has increased rapidly; specifically, CNNs can be considered a special type of multi-layer neural network that was built to directly identify visual patterns in pixel images with minimal preprocessing. CNNs have many benefits, such as an ability to extract more significant features from images rather than handcrafted features [20]. Researchers have proposed different CNN-based deep networks for achieving image classification [21,22], image segmentation [23], object detection, and localization in computer vision [24,25,26]. Besides solving natural computer vision problems, CNNs have also been very successful and efficient in solving medical problems, such as breast cancer detection [27], brain tumor segmentation [28], diagnosing Alzheimer’s disease, and classifications of skin lesions [29,30]. In addition, detailed reviews about deep learning in medical image analysis have been presented [31,32]. Various CNN models, such as ResNet, AlexNet, LeNet, VGGNet, and Inception were developed as pre-trained models on millions of images and can be used for image classification using transfer learning. These models have disadvantages—a very large architecture, millions of trainable parameters that require substantial computing power, and high time consumption [26]. Moreover, when the used dataset size is small, these models may overfit the training data, resulting in poor classification accuracy.

### 3.2. Recurrent Neural Networks (RNN)

Recurrent Neural Networks (RNNs) are the other type of deep learning technique and are mainly used for prediction purposes. They feed the output from the previous step and use it as an input for the current step. In this case, the networks themselves have repetitive loops. These loops, which are in the hidden neurons, allow for the storing of previous input information for a while so that the system can predict future outputs. Its most important feature is the hidden state, which remembers information about the sequence. They are also powerful tools for obtaining healthier modeling and prediction performance. The problem is that, when the network contains a large number of deep layers, they become untrained, which is called the vanishing gradient problem [33].

### 3.3. Long Short-Term Memory (LSTM)

One of the most famous types of RNN is the Long Short-Term Memory (LSTM) technique, which can be used mainly for large neural networks. The main benefit of the LSTM is that it can model both short- and long-term memory and can address the disappearance of the vanishing gradient problem that appears in RNNs by training on long strings and keeping them in memory [33]. These are the main types of deep learning techniques. These motivated us to build a CNN and LSTM combination architecture that helps to extract features and image classification, using the advantages of both types.

### 3.4. Pre-Trained Convolutional Neural Networks

There are two well-known pre-trained deep learning methods based on CNNs: ResNet152v2 and MobileNetv2 [34]. These models have many applications, such as classification, feature extraction, and prediction.

- ResNet152v2 Architecture
  Residual Network (ResNet) is a CNN architecture with hundreds or thousands of convolutional layers. Previous CNN structures decreased the efficacy of additional layers. ResNet contains a huge number of layers, with strong performance [34]. The primary difference between ResNetV2 and the original (V1) is that V2 uses batch normalization before each weight layer. In the field of image recognition and localization tasks, ResNet has strong performance that demonstrates the importance of many visual recognition tasks.
- MobileNetV2 Architecture
  The architecture of MobileNetV2 is based on an inverted residual structure where the shortcut connections of the residual block are between the thin bottleneck layers. The intermediate expansion layer of the MobileNetV2 uses lightweight depth-wise convolutions in order to filter the features. In traditional residual models, expanded representations in the input are used [34]. MobileNetV2 consists of the primary full convolution layer through 32 filters, followed by 19 residual bottleneck layers.

## 4. The Deep-Pneumonia Framework

**Figure 1:**
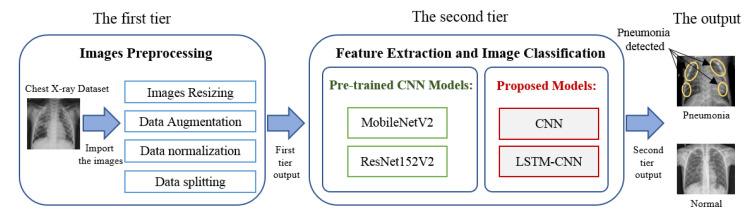
The proposed deep learning framework for pneumonia diagnosis.

As seen in the literature, there have been many deep learning models introduced to diagnosis pneumonia from chest X-ray images. These models introduce various values in performance metrics to verify the model validation. One of the extreme challenges has been to find an appropriate and efficient model that meets all performance metrics. The objectives of our study are (i) to propose a deep learning framework for pneumonia classification with four different models, and (ii) to evaluate the proposed models by comparing them with different recently introduced models. A deep learning framework for pneumonia diagnosis was developed, as shown in Figure 1. Our model has mainly two tiers. The first tier is responsible for image pre-processing, such as resizing, augmentation, data splitting, and data normalization. Data normalization is used for re-scaling the image’s pixel value to the interval [0,1]. The second tier works on feature extraction and image classification using different types of deep learning models.

The first tier includes image pre-processing, such as resizing, augmentation, data splitting, and normalization. The images are resized to 224×224×3. For increasing the number of training images to produce efficient and reliable pneumonia diagnosis systems, data augmentation techniques are used, such as rotate, flip, and skewing. The second tier starts by using the pre-processed image as its input, with a size of 224×224×3, followed by the deep learning model for feature extraction and image classification.

## 5. The Proposed Architectures

In this framework, four different types of supervised deep learning models are developed: CNN, LSTM-CNN, Resenet152V2, and MobilenetV2.

### 5.1. CNN Model

**Figure 2:**
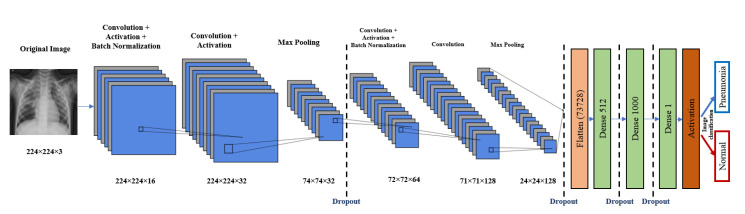
Proposed CNN architecture.

Deep neural networks with convolutional neural networks (CNNs) are employed to identify the pneumonia diagnosis of chest X-rays as a feature extraction and classification method. The proposed CNN model is demonstrated in Figure 2. It consists of input, feature extraction, and classification layers.

**Figure 3:**
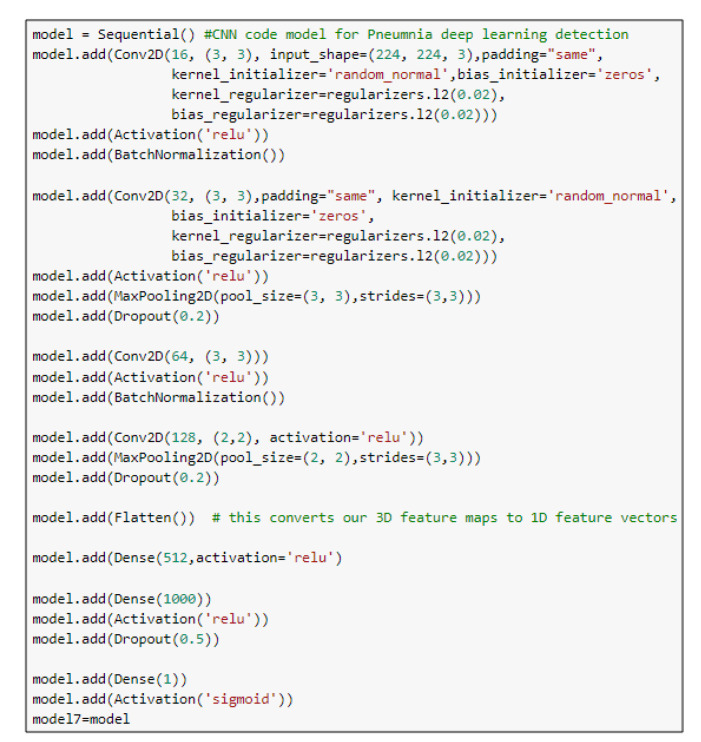
The main function code of the CNN model.

The input layer has a 224×224×3 chest image. The feature extraction part consists of four CNN blocks. Each one of these blocks has mainly a convolution layer, a batch normalization layer, and a ReLU layer. It may have maximum pooling and a dropout layer, as shown in Figure 2. The output of the feature extraction part is then passed to the flattened layer to change the data shape to a one-dimensional data vector, which is the correctly used format for the classification dense layer. The dense layer is the regular, deeply connected neural network layer. It is the most common and frequently used layer, where every input is connected to every output [34]. Here, we use three dense layers and four dropout layers. The final output is produced from a dense layer with sigmoid activation function that classifies the output image to Pneumonia (represented in the figure by blue arrow) or normal (represented by red arrow). The proposed CNN model architecture is listed in Table 2, and the main function of the code is shown in Figure 3. The total number of model parameters is 38,320,049: the trainable parameters amount to 38,319,889, and the non-trainable parameters only amount to 160.

### 5.2. The LSTM-CNN Model

**Figure 4:**
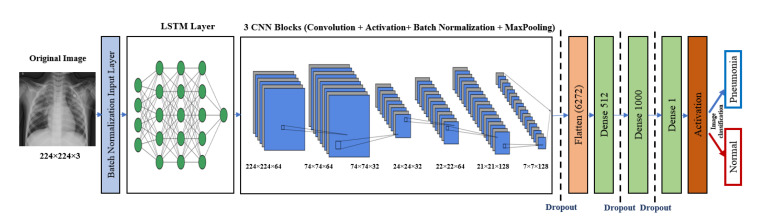
Proposed LSTM-CNN architecture.

**Figure 5:**
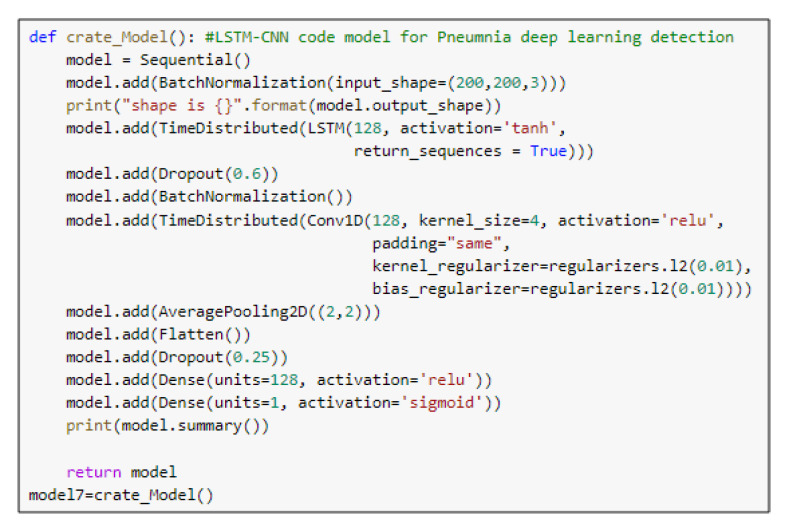
The main function code of the LSTM-CNN model.

LSTM is one of the recurrent neural network (RNN) architectures. LSTM has the ability of an RNN in modeling time series. A combination of LSTM and CNN was introduced in [35]. The LSTM-CNN proposed model is shown in Figure 4. As Figure 4 illustrates, a batch normalized layer is used before LSTM to prepare the input for LSTM. Time distribution is used with LSTM and the first CNN layers to change the images into time series data that are suitable for the LSTM structure, and this is followed by four CNN blocks, each of which basically has a convolutional layer and a batch normalization layer, and some blocks also have pooling and dropout layers. This part is for the feature extraction. The classification part consists of a flattened layer, two blocks of dense dropout layers, and a dense output layer with sigmoid activation function that classifies the output image to Pneumonia (represented in the figure by blue arrow) or normal (represented by red arrow). The proposed LSTM-CNN model architecture is demonstrated in Table 3, and the main function of the LSTM-CNN code is shown in Figure 5. The total number of parameters is 3,825,917: the trainable parameters amount to 3,825,655, and the non-trainable parameters only amount to 262.

### 5.3. Pre-Trained Models

**Figure 6:**
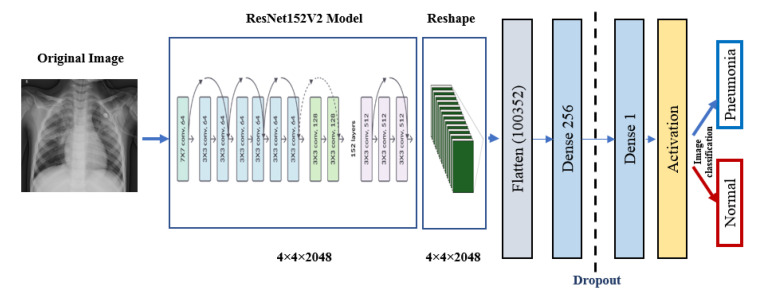
ResNet152V2 architecture.

**Figure 7:**
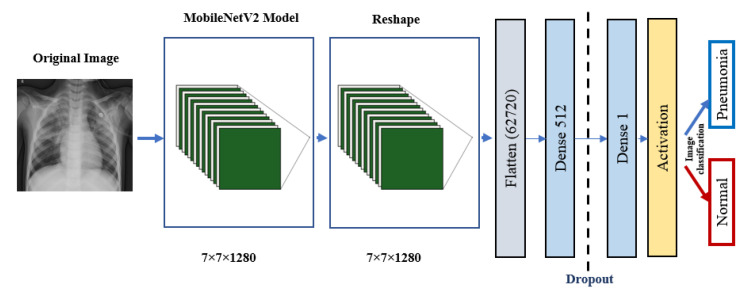
MobileNetV2 architecture.

**Figure 8:**
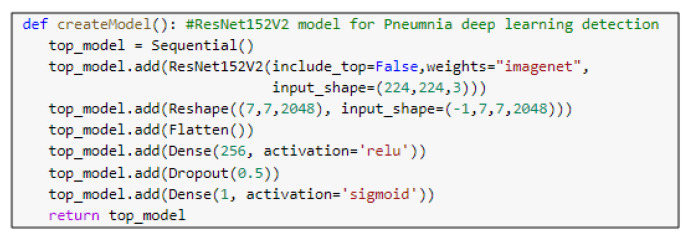
The main function code of the ResNet152V2 model.

**Figure 9:**
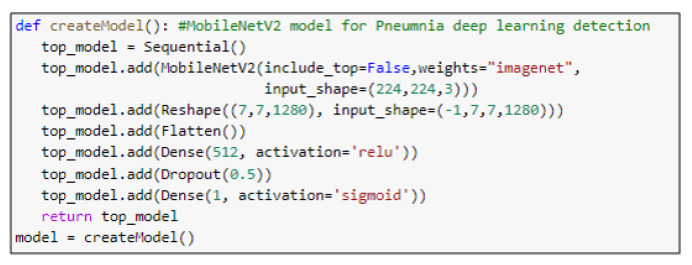
The main function code of the MobileNetV2 model.

ResNet152V2 and MobileNetV2 are also used as feature extraction models, as shown in Figure 6 and Figure 7, respectively. These models can train the input based on their pre-trained initial weights. This approach accelerates the training and coverage to high accuracy. Each model architecture contains the original model followed by a reshape step, flatten step, first dense layer, a dropout layer, second dense layer, and finally an activation function that classify the image to Pneumonia or normal represented in the figure by blue and red arrows, respectively. Their architectures are illustrated in Table 4 and Table 5, respectively. The main functions of the ResNet152V2 and MobileNetV2 codes are shown in Figure 8 and Figure 9, respectively. The total parameters of the ResNet152V2 amount to 84,022,273: the trainable parameters amount to 83,878,529, and the non-trainable parameters amount to 143,744. For the MobileNetV2, they amount to 34,371,649: 34,337,537 and 34,112 trainable and non-trainable parameters, respectively.

## 6. Methodology

### 6.1. Dataset

**Figure 10:**
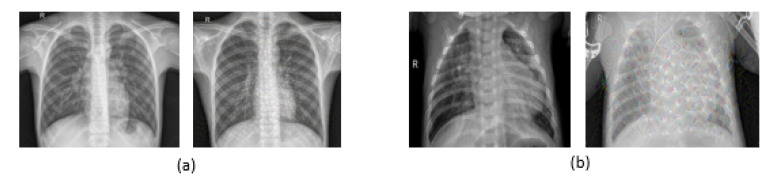
Chest X-ray images: (**a**) Normal images with and without a deep dream filter; (**b**) pneumonia images with and without a deep dream filter.

In our work, a publicly available Pneumonia Detection dataset of chest X-rays in Kaggle [36] was used, which consists of a total of 5856 images captured by a digital computed radiography (CR) system. Approximately 1583 of them are normal, and 4273 indicate pneumonia (65% for bacterial pneumonia and 35% for viral pneumonia). As shown in Figure 10, samples of chest X-ray images for normal cases and pneumonia cases are shown with different characteristics, such as Deep Dream filter. These images were augmented to increase the number of images for each classification—30,855 images: 8353 normal images and 22,502 pneumonia images. The patients’ ages in this dataset were divided into four ranges: about 6.5% of the patients were <20 years old, 26.4% were between 20 and 40, 42.8% were between 40 and 60, and 24.3% were >60. Regarding the patient’s gender in the dataset, 44% of the images represent female cases, and 56% represent male cases [36]. The images in the dataset range have resolutions ranging from 712×439 pixels to 2338×2025 pixels. Before inputting the images into the models, we downscaled the images to 224×224. In our framework, the training images are divided as follows: 70% for training and 30% for validation. The sample images were randomly split into two parts (train and validation).

### 6.2. The Used Deep-Pneumonia Platform

**Figure 11:**
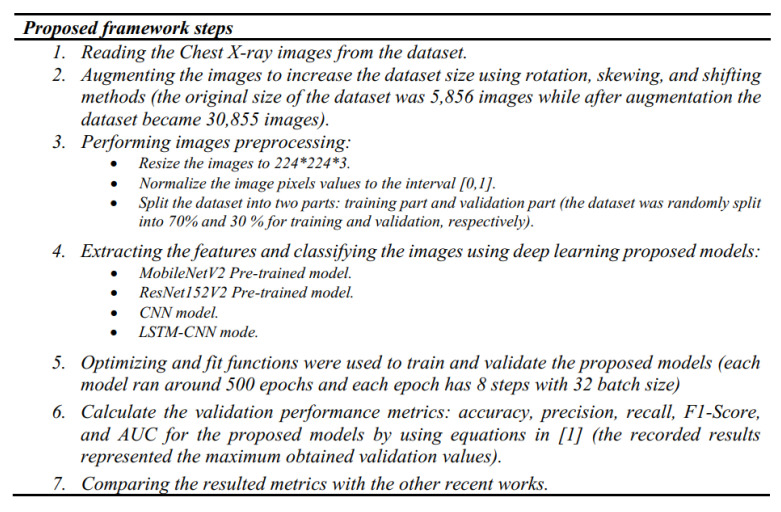
The proposed Deep-Pneumonia framework steps.

Our models were run using the pro version of Google Colab [37], which has 200 GB for storage, 25 GB RAM, and a P100 Graphical Processing Unit (GPU) processor. To obtain the statistical results, the pneumonia images were augmented using an Augmentor API in Keras [37] by image rotation, skew, and shift, and resizing and normalization were applied. The resulting images were fed into our deep learning models. The optimizer and fit functions were used to train and validate these models, where each model ran around 500 epochs and each epoch has 8 steps with a batch size of 32. The results were obtained by applying the equations for each performance metric [1] to the resulting validation data outputs and the recorded results represented the maximum obtained validation values. The complete code for our deep learning framework models was uploaded to the GitHub website in [38]. The proposed framework steps are summarized in Figure 11.

The performance of the deep learning system was evaluated based on such matrices as Loss, Accuracy, Precision, F1-score, Recall (Sensitivity), and Area Under the Curve (AUC) [1].

## 7. Experimental Results and Discussion

To study the performance of the pneumonia diagnosis with deep learning frameworks, the Python programming language with the help of Keras [34] was used for framework implementation. Google Colab [37] was used in the GPU runtime in the training and validation phases. ReLU and sigmoid activation functions were used for the hidden layers and the output layer, respectively. The number of epochs changed from 200 to 300 epochs based on the type of model, and the batch size was 32 for both the training and validation parts. The optimizer used for the pre-trained models is the Stochastic gradient descent (SGD) optimizer while the Adamax optimizer is used for the CNN and LSTM-CNN models. Finally, the learning rate (LR) and network parameters for all models are listed in Table 6.

Table 7 illustrates the validation metric values for the proposed models: ResNet152V2, MobileNetV2, CNN, and LSTM-CNN. The accuracy, precision, F1-score, and recall were calculated.

A comparison between the proposed completed work of this paper and the validation results of the other recent works introduced based on the same chest X-ray dataset is illustrated in Table 8. Research R1, R2, and R7 reported only one performance metric: AUC, F1-score, and accuracy, respectively. Accuracy, recall, and AUC were measured in R3. Only accuracy and recall were calculated in R4. The authors in R5 focused on accuracy, recall, and precision. R6 calculated recall, F1-score, and precision. Only one piece of research, R8, reported all five validation metrics. As clearly shown in Table 8, it is evident that our proposed ResNet152V2 model achieves the highest results for all used performance metrics in comparison with these previous works—represented by the bold numbers in the table. Additionally, all our proposed models exceed the recently introduced methods in the literature.

**Figure 12:**
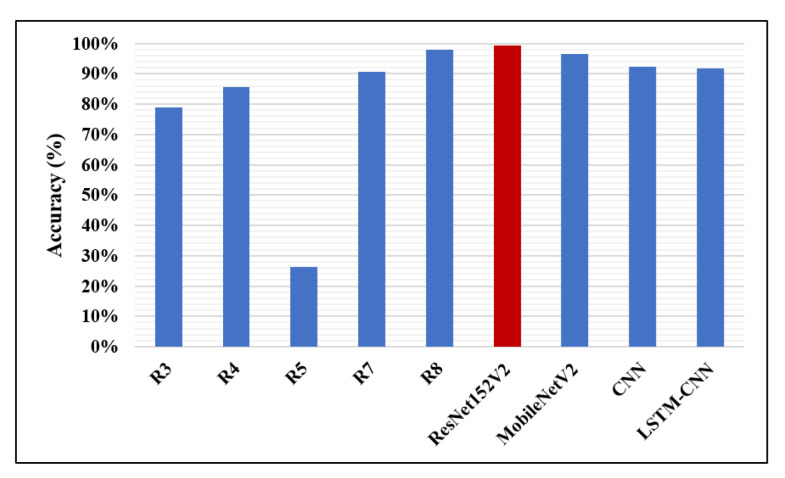
Accuracy performance metric.

**Figure 13:**
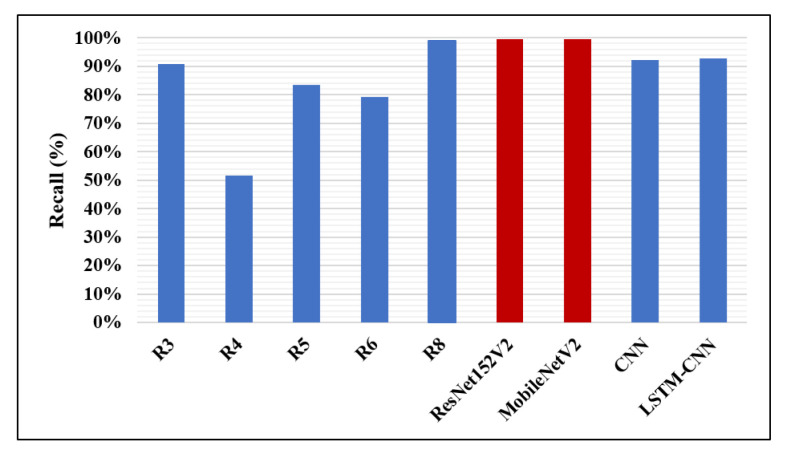
Recall performance metric.

The accuracy percentages of detecting pneumonia using CNN models in the recent works and the models presented in this work are illustrated in Figure 12. It is clear that our ResNet152V2 model has the best accuracy value (99.22%) compared with the other research, the highest accuracy reported of which is 98%. In Figure 13, our two proposed models, ResNet152V2 and MobileNetV2, achieve the best recall values for pneumonia detection, with values of 99.44% and 99.43%, respectively, compared with the 99% reported in R8.

**Figure 14:**
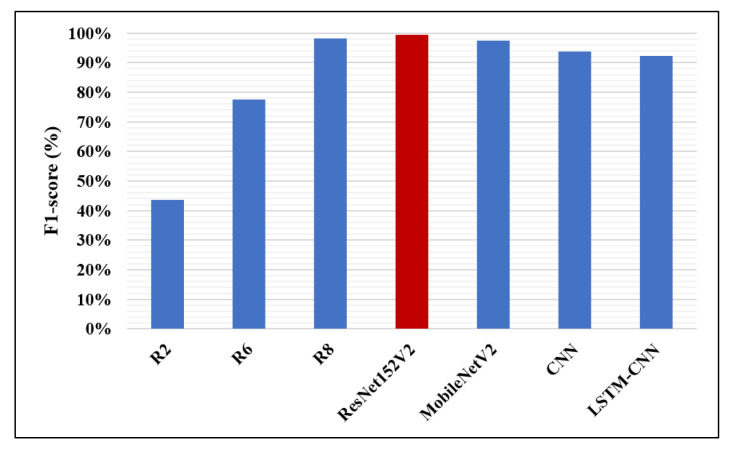
F1-score performance metric.

**Figure 15:**
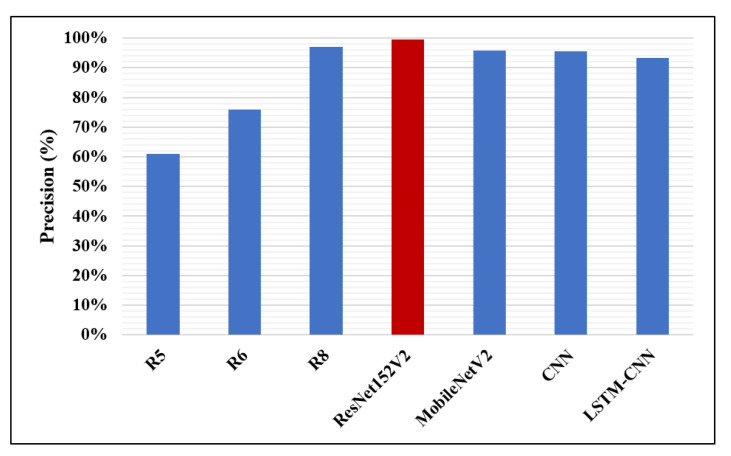
Precision performance metric.

**Figure 16:**
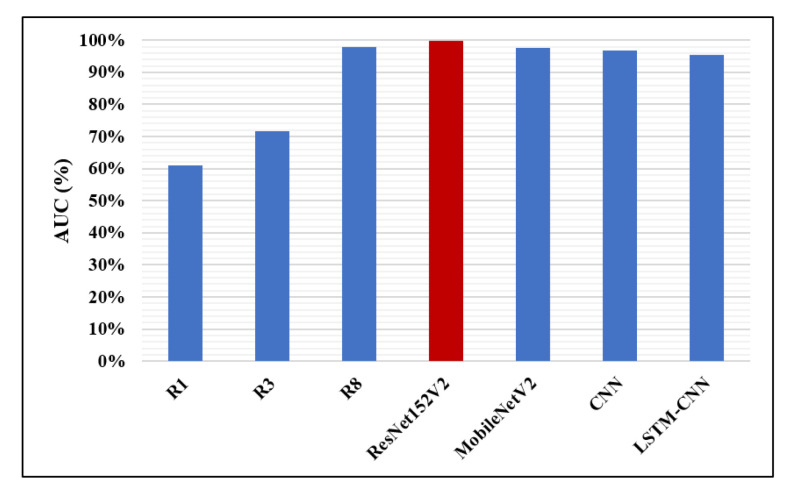
Area Under the Curve (AUC) performance metric.

Likewise, in Figure 14, Figure 15 and Figure 16, the F1-score, precision, and AUC are presented, respectively. Each of our proposed four models are compared against the previous, similar work. As clearly shown in the figures, the ResNet152V2 model obtains 99.44%, 99.44%, and 99.77% in F1-score, precision, and AUC, respectively. By comparing this with the 98.1%, 97%, and 98% values reported in the recent research, we conclude that the proposed ResNet152V2 model is the highest performing model. In addition to that, the other three proposed models achieve results with more than 90% in accuracy, recall, F1-score, precision, and AUC, which is superior to those of the other recently introduced models.

## 8. Conclusions and Future Work

In this paper, a deep learning framework for pneumonia classification with four different CNN models was proposed. Two of them were pre-trained models, ResNet152V2 and MobileNetV2, and the others were designed from scratch. We evaluated the proposed models by comparing them with recent, similar research. The experiment performance of our proposed deep learning framework was assessed based on accuracy, precision, F1-score, recall, and AUC, and our model showed values of 99.22%, 99.43%, 99.44%, 99.44%, and 99.77%, respectively. It is evident that our proposed ResNet152V2 model accomplished the highest results compared with the others. Moreover, the other three proposed models, MobileNetV2, CNN, and LSTM-CNN, achieved results of more than 91% in accuracy, recall, F1-score, precision, and AUC.

For future work, we plan to apply other CNNs and RNNs as bidirectional LSTM architectures and pre-trained models for detecting pneumonia using chest X-ray images.

## Figures and Tables

**Table 1:** Summary of recent work used in detecting pneumonia using a convolutional neural network (CNN).

Research	Author	Technique	Accuracy	Recall	F1-Score	Precision	AUC
R1	Antin B. et al. [12]	CNN+Adam (DenseNet121)	–	–	–	–	60.9%
R2	Rajpurkar P. et al. [13]	CNN (DensNet121)	–	–	43.5%	–	–
R3	Donthi A. et al. [15]	CNN	78.9%	90.7%	–	–	71.7%
R4	Almubarok A. et al. [16]	(deep ResNet), mask RCNN +Adam + FPN	85.60%	51.52%	–	–	–
R5	Li B. et al. [17]	CNN (RetinaNet, Mask R-CNN)	26.2%	83.5%	–	61.1%	–
R6	Sirazitdinov I. et al. [18]	CNN (RetinaNet, Mask R-CNN) + FPN principle	–	79.3%	77.5%	75.8%	–
R7	Sharma H. et al. [19]	CNN (4 models)	90.68%	–	–	–	–
R8	Rahman T. et al. [1]	AlexNet, ResNet18, DenseNet201, SqueezeNet	98%	99%	98.1%	97%	98%

AUC = Area Under the Curve.

**Table 2:** The proposed CNN model architecture.

Layer (Type)	Output Shape	Parameters
conv2d_37 (Conv2D)	(None, 224, 224, 16)	448
activation_37 (Activation)	(None, 224, 224, 16)	0
batch_normalization_19 (Batch)	(None, 224, 224, 16)	64
conv2d_38 (Conv2D)	(None, 224, 224, 32)	4640
activation_38 (Activation)	(None, 224, 224, 32)	0
max_pooling2d_19 (MaxPooling2d)	(None, 74, 74, 32)	0
dropout_37 (Dropout)	(None, 74, 74, 32)	0
conv2d_39(Conv2D)	(None, 72, 72, 64)	18,496
activation_39 (Activation)	(None, 72, 72, 64)	0
batch_normalization_20 (Batch)	(None, 72, 72, 64)	256
conv2d_40 (Conv2D)	(None, 71, 71, 128)	32,896
max_pooling2d_20 (MaxPooling2d)	(None, 24, 24, 128)	0
dropout_38 (Dropout)	(None, 24, 24, 128)	0
flatten_10 (Flatten)	(None, 73728)	0
dense_28 (Dense)	(None, 512)	37,749,248
dropout_39 (Dropout)	(None, 512)	0
dense_29 (Dense)	(None, 1000)	513,000
dropout_40 (Dropout)	(None, 1000)	0
dense_30 (Dense)	(None, 1)	1001
activation_40 (Activation)	(None, 1)	0

**Table 3:** The proposed LSTM-CNN model architecture.

Layer (Type)	Output Shape	Parameters
batch_normalization_7 (Batch)	(None, 224, 224, 3)	12
time_distribution_6 (TimeDistribution)	(None, 224, 224, 64)	17,408
time_distribution_6 (TimeDistribution)	(None, 224, 224, 64)	12,352
activation_9 (Activation)	(None, 224, 224, 64)	0
batch_normalization_8 (Batch)	(None, 224, 224, 64)	256
max_pooling2d_7 (MaxPooling2d)	(None, 74, 74, 64)	0
conv2d_7 (Conv2D)	(None, 74, 74, 32)	18,464
activation_10 (Activation)	(None, 74, 74, 32)	0
max_pooling2d_8 (MaxPooling2d)	(None, 24, 24, 32)	0
dropout_9 (Dropout)	(None, 24, 24, 32)	0
conv2d_8(Conv2D)	(None, 22, 22, 64)	18,496
activation_11 (Activation)	(None, 22, 22, 64)	0
batch_normalization_9 (Batch)	(None, 22, 22, 64)	256
conv2d_9 (Conv2D)	(None, 21, 21, 128)	32,896
max_pooling2d_9 (MaxPooling2d)	(None, 7, 7, 128)	0
dropout_10 (Dropout)	(None, 7, 7, 128)	0
flatten_3 (Flatten)	(None, 6272)	0
dense_7 (Dense)	(None, 512)	3,211,776
dropout_11 (Dropout)	(None, 512)	0
dense_8 (Dense)	(None, 1000)	513,000
dropout_12 (Dropout)	(None, 1000)	0
dense_9 (Dense)	(None, 1)	1001
activation_12 (Activation)	(None, 1)	0

**Table 4:** The pre-trained ResNet152V2 model architecture.

Layer (Type)	Output Shape	Parameters
resnet152v2 (Model)	(None, 4, 4, 2048)	58,331,648
reshape_2 (Reshape)	(None, 4, 4, 2048)	0
flatten_2 (Flatten)	(None, 100352)	0
dense_3 (Dense)	(None, 256)	25,690,368
dropout_2 (Dropout)	(None, 256)	0
dense_4 (Dense)	(None, 1)	257

**Table 5:** The pre-trained MobileNetV2 model architecture.

Layer (Type)	Output Shape	Parameters
mobilenetv2_1.00_224 (Model)	(None, 7, 7, 1280)	2,257,984
reshape_2 (Reshape)	(None, 7, 7, 1280)	0
flatten_2 (Flatten)	(None, 62720)	0
dense_3 (Dense)	(None, 512)	32,113,152
dropout_2 (Dropout)	(None, 512)	0
dense_4 (Dense)	(None, 1)	513

**Table 6:** Models’ training parameters.

Models		Optimizer	Learning Rate (LR)	Total Number of Parameters
Pre-trained models	ResNet152V2	SGD	0.0001	84,022,273
	MobileNetV2	SGD	0.0001	34,371,649
Our proposed models	CNN	Adamax	0.00003	38,320,049
	LSTM-CNN	Adamax	0.00006	3,825,917

SGD = Stochastic gradient descent

**Table 7:** The performance validation of the proposed models.

Models		Loss	Accuracy	Precision	AUC	F1-Score	Recall
Pre-trained models	ResNet152V2	0.0523	99.22%	99.44%	99.77%	99.44%	99.43%
	MobileNetV2	0.1665	96.48%	95.68%	97.50%	97.52%	99.44%
Our proposed models	CNN	0.3020	92.19%	95.57%	96.92%	93.79%	92.07%
	LSTM-CNN	0.5771	91.80%	93.24%	95.49%	92.29%	92.62%

**Table 8:** Comparison with related works.

Research	Author	Accuracy	Recall	F1-Score	Precision	AUC
R1	Antin B. et al. [12]	–	–	–	–	60.9%
R2	Rajpurkar P. et al. [13]	–	–	43.5%	–	–
R3	Donthi A. et al. [15]	78.9%	90.7%	–	–	71.1%
R4	Almubarok A. et al. [16]	85.60%	51.52%	–	–	–
R5	Li B. et al. [17]	26.2%	83.5%	–	61.1%	–
R6	Sirazitdinoy I. et al. [18]	–	79.3%	77.5%	75.8%	–
R7	Sharma H. et al. [19]	90.68%	–	–	–	–
R8	Rahman T. et al. [1]	98%	99%	98.1%	97%	98%
**The proposed** **four models**	**ResNet152V2**	**99.22%**	**99.43%**	**99.44%**	**99.44%**	**99.77%**
	**MobileNetV2**	96.48%	**99.44%**	97.52%	95.68%	97.50%
	**CNN**	92.19%	92.07%	93.79%	95.57%	96.92%
	**LSTM-CNN**	91.80%	92.62%	92.29%	93.24%	95.49%

Bold number: ResNet152V2 model achieves the highest results for all used performance metrics in comparison with these previous works.
